# Age as an Independent Predictor of MCID Achievement and Patient Satisfaction Following Elective Cervical Spine Surgery

**DOI:** 10.1177/21925682261508000

**Published:** 2026-11-11

**Authors:** Theodore A. Joaquin, Jacob W. McDevitt, Andre C. Ferreira, Cade F. Bennett, Tyler M. Compton, Daniel E. Herrera, Jacob R. Staub, Lidia Hrvojevic, Aaron K. Hoyt, Matthew W. Colman, Wellington K. Hsu, Srikanth N. Divi, Alpesh A. Patel

**Affiliations:** 1 Department of Orthopaedic Surgery, 12244 Northwestern University Feinberg School of Medicine, Chicago, IL, USA

**Keywords:** cervical spine surgery, minimal clinically important difference, PROMIS, patient-reported outcomes, age, patient satisfaction, preoperative expectations, expectation management

## Abstract

**Study Design:**

Retrospective Cohort Study.

**Objectives:**

To determine whether age independently predicts minimal clinically important difference (MCID) achievement in physical function (PF) and pain interference (PI) following elective cervical spine surgery, and to examine how age-related expectation-achievement discordance influences postoperative satisfaction.

**Methods:**

Adult patients undergoing elective cervical spine surgery at a single academic center (2022-2025) were included if they completed preoperative and 12-month Patient-Reported Outcomes Measurement Information System (PROMIS) PF and PI computerized adaptive tests (CATs) and a preoperative expected outcome assessment. MCID was defined as an 8-point PROMIS T-score change. Patients were stratified into five age groups (<45, 45-54, 55-64, 65-74, ≥75). Multivariable logistic regression modeled age continuously, adjusting for BMI, levels fused, sex, smoking, revision status, and pathology.

**Results:**

Among 129 patients, MCID achievement declined significantly with age for both domains (trend p<0.001). Each additional year of age independently reduced odds of achieving MCID (PF: OR 0.95, p=0.007; PI: OR 0.96, p=0.02). Adjusted MCID probability fell below 50% at 45.9 years for PF and 49.1 years for PI. The expectation-achievement gap was widest in middle-aged patients (45-64), who held expectations comparable to younger patients yet achieved outcomes consistent with older cohorts. Among non-achievers, satisfaction increased with advancing age (trend p=0.05 for PF).

**Conclusions:**

After adjusting for the covariates assessed, age continuously and independently predicts failure to achieve MCID after cervical spine surgery, with probability falling to 50% by the late forties. Despite this, older non-achievers report high satisfaction, suggesting MCID is not a universal prerequisite for surgical success and underscoring the need for age-specific preoperative counseling.

## Introduction

Degenerative cervical spine pathology afflicts all ages, most commonly arising in the fifth and sixth decades of life.^1^ Together, these cases represent one of the most common indications for elective spine surgery.^1-3^ The spectrum of cervical disease shifts with advancing age, with radiculopathy predominating in younger patients and myelopathy becoming increasingly prevalent in older cohorts.^4,5^ As surgical indications continue to expand and the population ages, understanding how patient age influences postoperative outcomes has become increasingly important in guiding surgical decision-making and preoperative counseling.

While increasing age has been identified in the broader orthopaedic literature as a risk factor for suboptimal outcomes, it remains unclear how this relationship extends to patient satisfaction following cervical spine surgery.^6-8^ Younger patients may outperform older patients on objective recovery metrics, yet older patients may operate with an adjusted benchmark for what constitutes a satisfying surgical result. Patient expectations are known to be a powerful determinant of postoperative satisfaction in spine surgery, and a mismatch between anticipated and actual outcomes has been identified as a potential driver of dissatisfaction.^9-12^ Whether this expectation-achievement relationship varies meaningfully with age has not been well established.

This study aimed to investigate the extent to which patient age influenced postoperative recovery and satisfaction following elective cervical spine surgery. Utilizing Patient-Reported Outcomes Measurement Information System (PROMIS) computerized adaptive tests (CATs) for physical function (PF) and pain interference (PI), patient outcomes were directly compared to preoperative expectations and established MCID thresholds. The impact of discordance between these metrics on postoperative satisfaction was also examined across age groups.

## Methods

### Study Design and Patient Population

A retrospective review of prospectively collected data was performed at a single tertiary academic medical center after institutional review board approval. All patients provided consent for data collection and storage. The study included adult patients (≥18 years) who underwent elective cervical spine surgery by one of four fellowship-trained surgeons between 2022 and 2025. Clinical data were collected and managed using REDCap electronic data capture tools.

### Inclusion and Exclusion Criteria

Patients were included if they completed preoperative baseline and 12-month postoperative Patient-Reported Outcomes Measurement Information System (PROMIS) Physical Function (PF) and Pain Interference (PI) computerized adaptive tests (CATs), as well as a preoperative expected outcome assessment. Patients were excluded if they were missing any required PROMIS scores, had diagnoses of trauma, malignancy, or infection, or lacked complete covariate data required for multivariable analysis. A complete-case analytic approach was used, with a final cohort of 129 patients.

### Data Collection and Outcome Measures

Patients were enrolled independently by a research fellow either in their preoperative bay prior to surgery or in the clinic following preoperative counseling with their treating surgeon. The language used in the expectation management and informed consent conversations between the treating surgeons and their patients were not standardized.

Baseline functional status and pain level were assessed with PROMIS PF and PI CATs. Patients were then guided through an additional PROMIS CAT oriented toward their expected status at one year following surgery. Patients were instructed to answer the questions regarding their functional status and pain level in line with their anticipated status following 12 months of rehabilitation. An example of a question a patient might see on their baseline CAT is “Are you able to do chores such as vacuuming or yard work?” with the answer options of “Without any difficulty, With a little difficulty, With some difficulty, With much difficulty, Unable.” The corresponding question on the expected CAT would read “12 months following surgery, will you be able to do chores such as vacuuming or yard work?” followed by the same answer choices.

Actual postoperative PROMIS PF and PI scores were obtained again at 12 months. Higher PF scores indicate improved physical function, whereas higher PI scores indicate greater pain interference. Accordingly, improvement was defined as an increase in PF and a decrease in PI at 12 months.

### MCID Definitions

Change in PROMIS scores was calculated as the difference between 12-month and baseline scores for PF, and as the inverse (baseline minus 12-month) for PI to ensure consistent directionality of improvement. Expected change was calculated analogously using anticipated 12-month scores. The minimal clinically important difference (MCID) was defined as 8 PROMIS T-score points for both PF and PI, consistent with prior cervical spine literature.^13^ Patients were classified as achieving MCID if their observed change met or exceeded this threshold, and as expecting MCID if their anticipated improvement met or exceeded this threshold.

### Age Stratification and Covariates

Patients were stratified into five age groups: <45, 45-54, 55-64, 65-74, and ≥75 years. Demographic and clinical variables included age, body mass index (BMI), number of cervical levels fused, baseline PROMIS scores, sex, smoking status (ever vs. never), revision surgery status, and underlying cervical pathology (myelopathy, radiculopathy, or both).

### Statistical Analysis

Continuous variables were reported as mean ± standard deviation and categorical variables as counts with percentages. Between-group comparisons across age strata were performed using one-way analysis of variance for continuous variables and chi-square tests for categorical variables. Within each age group, the proportions of patients expecting and achieving MCID were calculated with 95% Wilson score confidence intervals. Paired comparisons between expected and achieved MCID rates were assessed using McNemar tests. Trends across increasing age groups were evaluated using the Cochran-Armitage trend test.

Multivariable logistic regression models were constructed to identify factors associated with achieving MCID for PF and PI. Age was modeled as a continuous variable. Covariates included BMI, number of levels fused, sex, smoking status, revision surgery, and cervical pathology. Results were reported as odds ratios (ORs) with 95% confidence intervals. Model fit was assessed using McFadden’s pseudo-R^2^. Adjusted probabilities of achieving MCID across age were estimated using average marginal predictions, and corresponding confidence intervals were derived from bootstrap resampling. All analyses were performed in Python (version 3.13.9).

## Results

Patient demographics and baseline characteristics did not significantly differ between age groups except for the rates of myelopathy and radiculopathy. Patients in the oldest age group were more likely to experience myelopathy (70.6%, p<0.001), whereas patients in the youngest group were more likely to have radiculopathy (53.3%, p=0.009). Increasing age was associated with higher rates of myelopathy and lower rates of radiculopathy. There were no significant differences in preoperative Physical Function (PF) scores between age groups (p=0.2); however, there was significant variation in baseline Pain Interference (PI) scores between groups (p<0.001), with patients under 45 demonstrating the highest degree of preoperative impairment. Complete demographics are summarized in Table 1.

**Table 1.** Patient Demographics and Baseline Characteristics

​	<45	45-54	55-64	65-74	≥75	Overall	p-value
N	15	23	34	40	17	129	-
Age, years	39.9 ± 4.7	50.2 ± 3.2	60.0 ± 2.9	70.8 ± 2.7	79.6 ± 3.8	61.8 ± 12.6	<0.001
Female sex, n (%)	9 (60.0%)	6 (26.1%)	15 (44.1%)	18 (45.0%)	5 (29.4%)	53 (41.1%)	0.22
BMI, kg/m^2^	28.5 ± 4.7	29.3 ± 5.7	27.6 ± 5.4	28.8 ± 5.8	28.1 ± 3.3	28.4 ± 5.2	0.82
Levels fused	1.9 ± 1.0	2.0 ± 1.8	2.2 ± 1.3	2.4 ± 1.5	2.7 ± 1.9	2.3 ± 1.5	0.48
Ever smoker, n (%)	5 (33.3%)	6 (26.1%)	12 (35.3%)	23 (57.5%)	8 (47.1%)	54 (41.9%)	0.11
Revision surgery, n (%)	1 (6.7%)	4 (17.4%)	5 (14.7%)	3 (7.5%)	3 (17.6%)	16 (12.4%)	0.65
Myelopathy, n (%)	2 (13.3%)	8 (34.8%)	18 (52.9%)	30 (75.0%)	12 (70.6%)	70 (54.3%)	<0.001
Radiculopathy, n (%)	8 (53.3%)	11 (47.8%)	9 (26.5%)	7 (17.5%)	2 (11.8%)	37 (28.7%)	0.009
Both, n (%)	5 (33.3%)	4 (17.4%)	6 (17.6%)	2 (5.0%)	3 (17.6%)	20 (15.5%)	0.12
Baseline PROMIS-PF	34.6 ± 5.4	38.7 ± 6.2	39.5 ± 7.5	38.1 ± 7.8	40.3 ± 8.5	38.5 ± 7.4	0.20
Baseline PROMIS-PI	69.4 ± 5.3	64.3 ± 6.2	64.0 ± 7.6	60.5 ± 8.4	56.6 ± 9.9	62.6 ± 8.4	<0.001

Data presented as mean ± SD for continuous variables and n (%) for categorical variables. p-values for continuous variables were calculated using one-way ANOVA; p-values for categorical variables were calculated using the chi-square test.

At 12 months, there were significant differences in the rates of achieving and expected MCID between the five groups. Younger patients both expected and achieved greater improvements in PF and PI scores. A higher percentage of patients in the <45 age group achieved MCID (PF: 60.0% | PI: 73.3%, p<0.001), though expectations for MCID were higher as well. A smaller proportion of patients in the ≥75 age group achieved MCID (PF: 11.8% | PI: 17.6%, p<0.001), though this age group also had lower baseline expectations for improvement. The proportion of patients meeting achieved and expected MCID thresholds declined with age (p<0.001). Group-level comparisons demonstrated widening gaps between expected and achieved MCID with increasing age, particularly in middle age groups (PF: p=0.04 for ages 55–64; p=0.004 for ages 65–74; PI: p=0.004 for ages 45–54; p=0.007 for ages 55–64). Achieved and expected MCID results are summarized in Table 2 and Figure 1. Mean PROMIS T-score change declined across age groups for both domains, falling below the MCID threshold of 8 points by the 45-54 group for PF and the 55-64 group for PI, and approaching zero in the ≥75 cohort. These trends are illustrated in Figure 2.

**Table 2.** Expected vs Actual MCID Achievement by Age Group

Domain	Age Group	N	Expecting MCID (%)	Achieving MCID (%)	McNemar p
PROMIS-PF	<45	15	80.0% (54.8-93.0)	60.0% (35.7-80.2)	0.45
PROMIS-PF	45-54	23	82.6% (62.9-93.0)	52.2% (33.0-70.8)	0.04
PROMIS-PF	55-64	34	55.9% (39.5-71.1)	32.4% (19.1-49.2)	0.04
PROMIS-PF	65-74	40	55.0% (39.8-69.3)	22.5% (12.3-37.5)	0.004
PROMIS-PF	≥75	17	35.3% (17.3-58.7)	11.8% (3.3-34.3)	0.12
Trend p-value	-	-	0.001	<0.001	-
PROMIS-PI	<45	15	86.7% (62.1-96.3)	73.3% (48.0-89.1)	0.69
PROMIS-PI	45-54	23	87.0% (67.9-95.5)	47.8% (29.2-67.0)	0.004
PROMIS-PI	55-64	34	67.6% (50.8-80.9)	35.3% (21.5-52.1)	0.007
PROMIS-PI	65-74	40	42.5% (28.5-57.8)	32.5% (20.1-48.0)	0.39
PROMIS-PI	≥75	17	41.2% (21.6-64.0)	17.6% (6.2-41.0)	0.22
Trend p-value	-	-	<0.001	<0.001	-

MCID Threshold 8. McNemar test for within-group paired comparisons, Cochran-Armitage test for trends.

**Figure 1.**
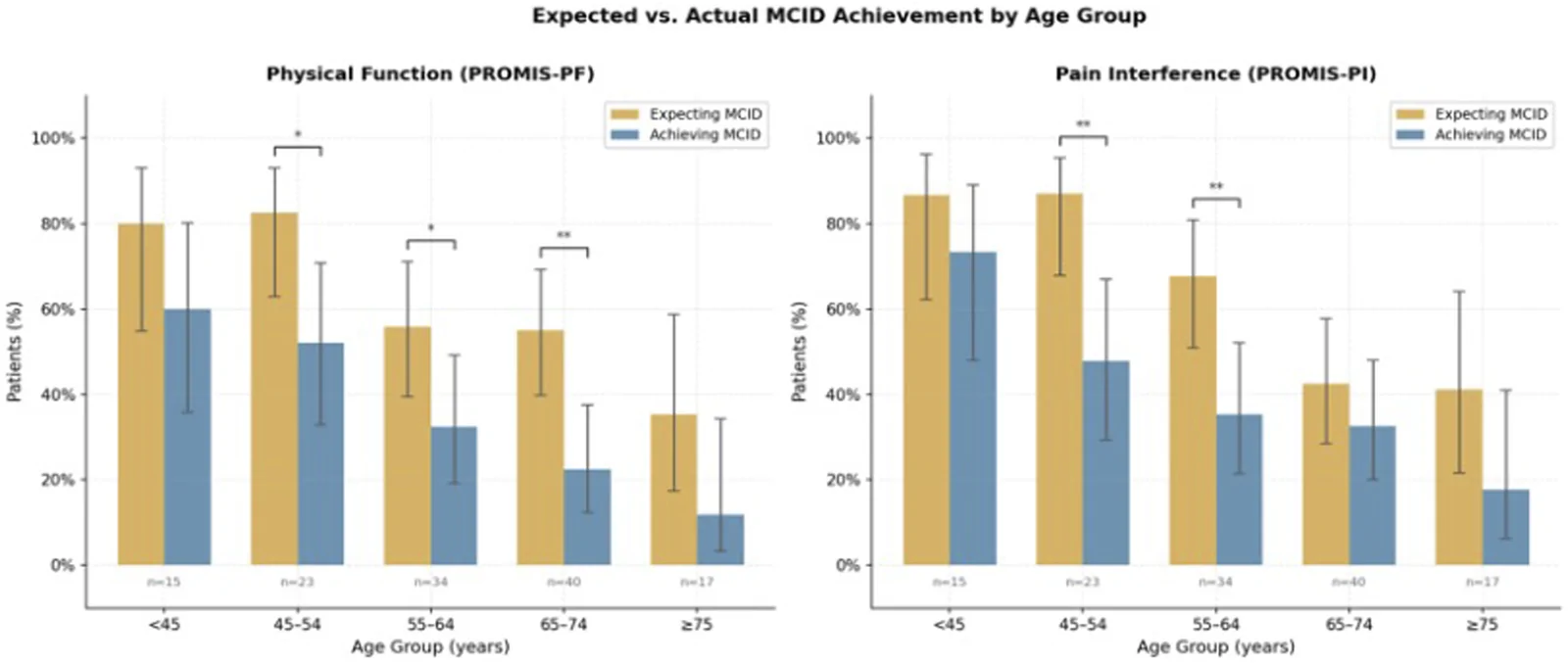
Achieving MCID vs Expecting MCID by age. Error bars represent 95% Wilson score confidence intervals. Significance brackets denote within-group McNemar test results comparing expected vs. achieved rates. *p<0.05, **p<0.01

**Figure 2.**
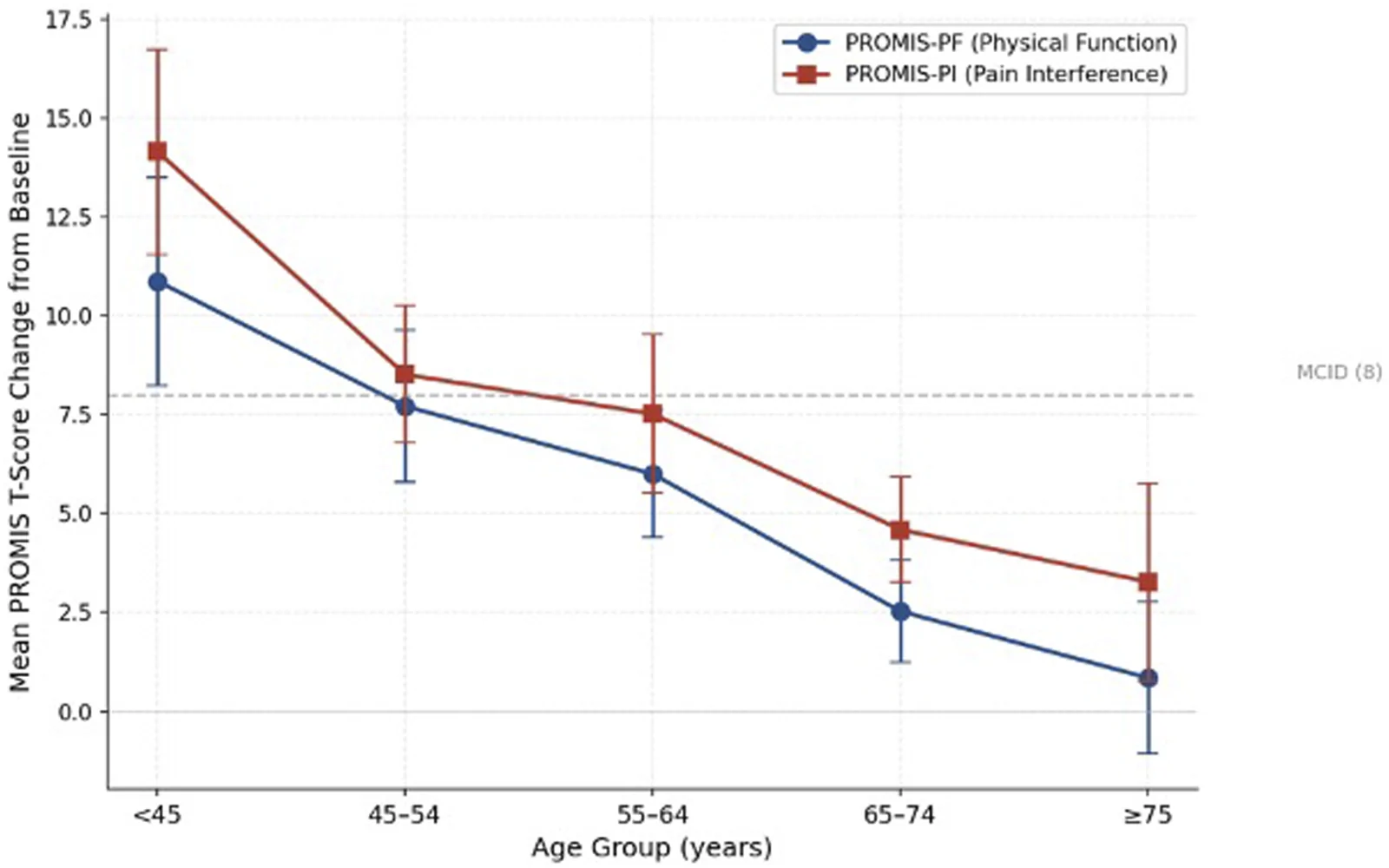
Mean PROMIS Score Change by Age Group. PI sign was flipped to match PF and show improvement trends. Error bars represent standard error of the mean

After controlling for differences in baseline characteristics and modeling age as a continuous variable, each additional year of age decreased the odds of achieving MCID (PF: OR 0.95, 95% CI 0.92–0.99, p=0.007 | PI: OR 0.96, 95% CI 0.93–0.99, p=0.02). The adjusted probability of achieving MCID declined continuously with age for both domains. The probability fell below 50% at 45.9 years for PF and 49.1 years for PI, as illustrated in Figure 3. A summary of age-related associations is shown in Table 3.

**Figure 3.**
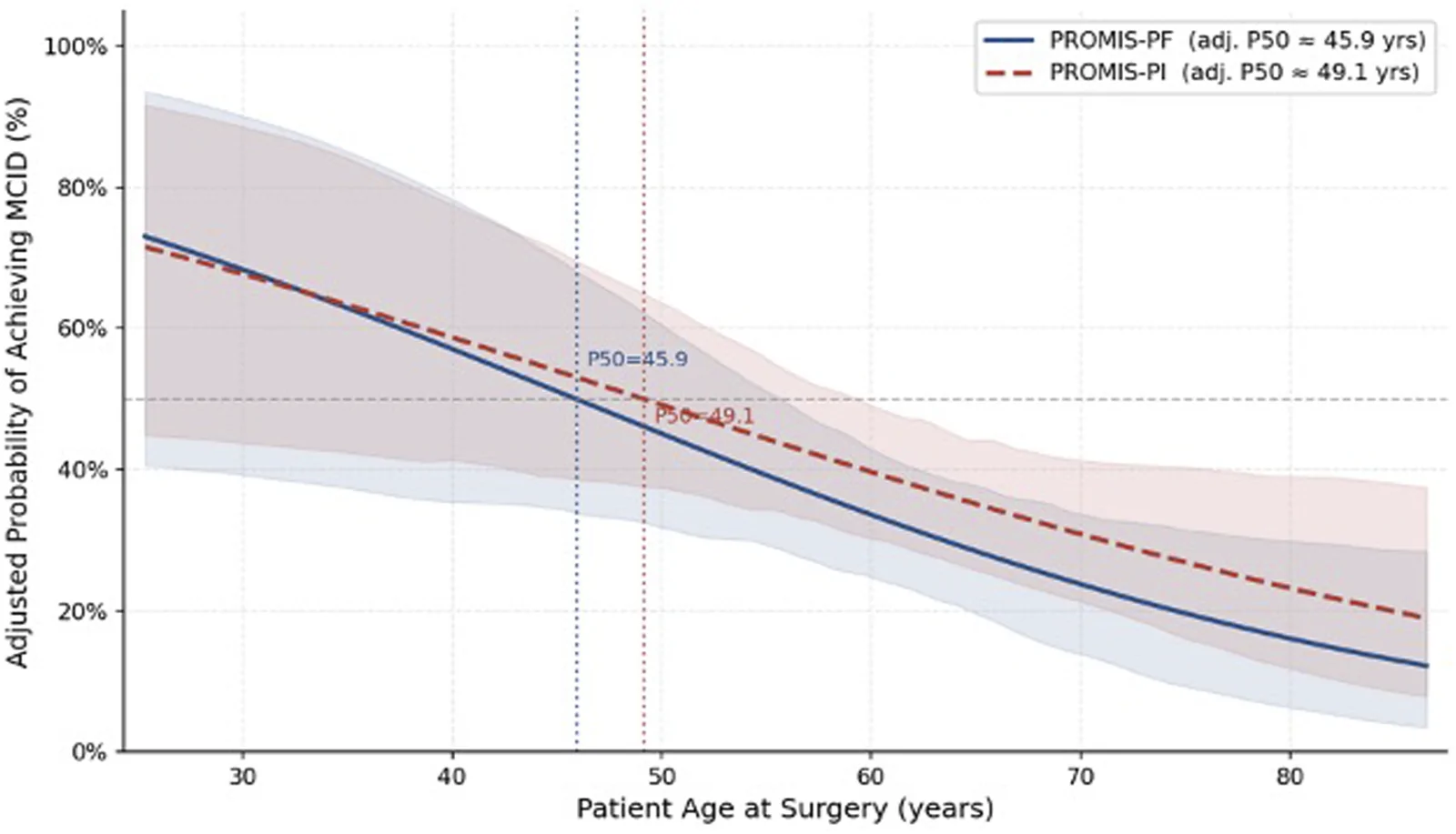
Adjusted Probability of Achieving MCID by Age - Multivariable Regression. Adjusted probabilities were calculated using average marginal predictions. For each age value, every patient's predicted probability was calculated using their own observed covariate values with age set to that value, then averaged across the cohort. Covariates included in both models: BMI, number of levels fused, sex, smoking status, revision surgery, and pathology. Shaded bands represent 95% bootstrap confidence intervals (500 resamples). The horizontal dashed line marks the 50% probability threshold; the adjusted P50 (age at which probability of achieving MCID falls to 50%) for each domain is in the legend

**Table 3.** Multivariable Logistic Regression: Predictors of MCID Achievement

Domain	Variable	β	SE	OR (95% CI)	p-value
PROMIS-PF	Age (per year)	-0.050	0.018	0.95 (0.92, 0.99)	0.007
PROMIS-PF	BMI (per unit)	0.004	0.039	1.00 (0.93, 1.08)	0.93
PROMIS-PF	Levels fused (per level)	-0.062	0.148	0.94 (0.70, 1.26)	0.68
PROMIS-PF	Female sex	0.192	0.421	1.21 (0.53, 2.77)	0.65
PROMIS-PF	Ever smoker	0.093	0.436	1.10 (0.47, 2.58)	0.83
PROMIS-PF	Revision surgery	0.327	0.625	1.39 (0.41, 4.72)	0.60
PROMIS-PF	Radiculopathy (vs. Myelopathy)	0.672	0.487	1.96 (0.75, 5.09)	0.17
PROMIS-PF	Both (vs. Myelopathy)	0.590	0.584	1.80 (0.57, 5.67)	0.31
PROMIS-PI	Age (per year)	-0.041	0.018	0.96 (0.93, 0.99)	0.02
PROMIS-PI	BMI (per unit)	-0.022	0.038	0.98 (0.91, 1.05)	0.57
PROMIS-PI	Levels fused (per level)	0.095	0.139	1.10 (0.84, 1.44)	0.49
PROMIS-PI	Female sex	-0.245	0.412	0.78 (0.35, 1.75)	0.55
PROMIS-PI	Ever smoker	0.150	0.420	1.16 (0.51, 2.65)	0.72
PROMIS-PI	Revision surgery	0.199	0.598	1.22 (0.38, 3.94)	0.74
PROMIS-PI	Radiculopathy (vs. Myelopathy)	0.838	0.472	2.31 (0.92, 5.83)	0.08
PROMIS-PI	Both (vs. Myelopathy)	0.923	0.568	2.52 (0.83, 7.66)	0.10

Reference category for pathology is myelopathy. 95% confidence intervals are Wald-type. Model fit is reported as McFadden’s pseudo-R^2^.

Postoperative satisfaction was assessed by age group and MCID achievement status and is summarized in Figure 4. Among patients who achieved MCID, satisfaction rates were uniformly high across all age groups for both PF and PI. Among patients who did not achieve MCID, satisfaction rates were lowest in the youngest age groups and increased with advancing age, with a statistically significant trend observed for PF non-achievers (trend p=0.05) and a similar but non-significant trend for PI non-achievers (trend p=0.17). In the youngest cohort, satisfaction rates were significantly higher among MCID achievers compared to non-achievers for PF (p<0.05). This divergence between objective outcome and subjective satisfaction diminished with advancing age, such that older non-achievers reported satisfaction rates comparable to their achieving counterparts.

**Figure 4.**
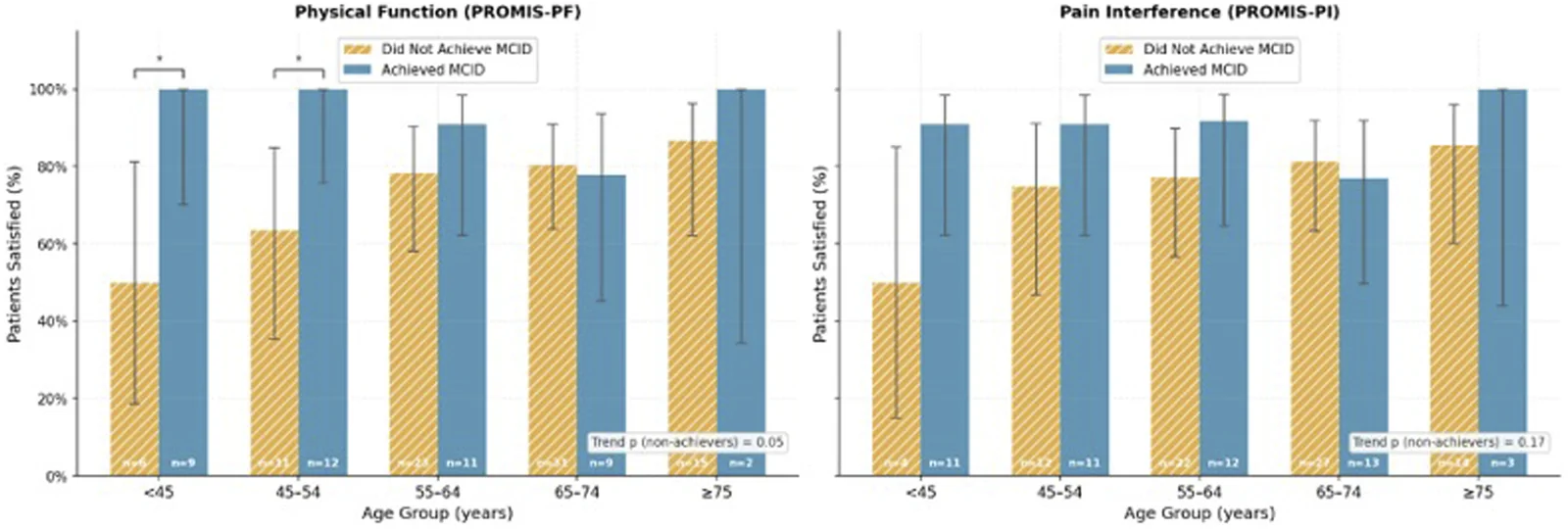
Satisfaction with Surgical Results by Age Group and MCID Achievement. Percentage of patients satisfied with surgical results stratified by age group and MCID achievement status for PROMIS Physical Function (left) and Pain Interference (right). Blue bars represent patients who achieved MCID; orange bars represent those who did not. Error bars denote 95% confidence intervals. Asterisks indicate statistically significant differences between achievers and non-achievers within an age group. Trend p-values reflect Cochran-Armitage testing for monotonic trends in satisfaction across age groups among non-achievers. MCID threshold = 8 PROMIS T-score points

## Discussion

In this study, patient age was inversely associated with the likelihood of achieving MCID in PF and PI following elective cervical spine surgery, independent of the covariates included in our adjusted models. Each additional year of patient age was associated with a 5% reduction in the odds of achieving MCID for PF and a 4% reduction for PI after controlling for BMI, surgical complexity, sex, smoking status, revision status, and underlying cervical pathology. Additionally, the probability of achieving MCID fell below 50% at approximately 46 years for PF and 49 years for PI. Interestingly, this decreased ability to achieve MCID in older patients was not accompanied by lower levels of patient satisfaction postoperatively. Older patients who failed to meet MCID reported comparable or greater satisfaction compared to their younger counterparts. These findings challenge the universality of MCID as a surgical success metric and demonstrate the need for age-informed preoperative counseling and expectation management.

Age has been identified as a risk factor for adverse and suboptimal outcomes following surgery across many different orthopaedic subspecialties.^6,14-16^ The continuously inverse relationship between patient age and MCID achievement observed in this study is consistent with current spine literature.^17,18^ Interestingly, age remained a significant independent predictor of MCID achievement after adjusting for cervical pathology. The persisting significance is a critical finding given that myelopathy disproportionately affects older individuals, even as radiculopathy predominates in younger age groups.^4,5^ This demonstrates that age influences surgical outcomes beyond its association with particular pathology types, potentially reflecting increased frailty, greater comorbidity burden, declining bone quality, and increased susceptibility to postoperative medical complications.^19-21^ Additionally, mean PROMIS T-score change fell below MCID by the 45-54 age group for PF and the 55-64 group for PI, suggesting that the majority of patients over 55 are unlikely to achieve clinically meaningful improvement.

Despite the elderly being least likely to achieve MCID, the expectation-achievement gap was widest for middle-aged patients (45-64). Young patients (<45) entered surgery with the highest expected outcomes, but also achieved MCID most consistently. On the other hand, patients over the age of 75 seemed to have properly tempered expectations. While they were least likely to achieve MCID, they did not anticipate the drastic recoveries that younger patients did and had relative alignment between anticipated and actual outcomes. Middle-aged patients, by contrast, approached surgery with expectations commensurate with younger patients yet achieved outcomes more consistent with older cohorts. Current literature has identified expectation fulfillment as one of the strongest predictors of postoperative satisfaction in spine surgery.^9-12^ These studies emphasize unmet expectations, rather than poor absolute outcomes, as primary drivers of the patients’ opinions of surgical success. The present findings suggest that middle-aged patients represent a high-risk group for unmet expectations and warrant targeted preoperative counseling and expectation-setting.

Since the older population of patients were less likely to meet MCID, it would intuitively follow that they would also be less satisfied. However, the satisfaction analysis identified a paradoxical relationship between patient outcomes and satisfaction. Among MCID non-achievers, satisfaction rates were lowest in the youngest patients and increased with advancing age, reaching a statistically significant trend for physical function (trend p=0.05, Figure 4). Despite lower rates of objective MCID achievement, older patients who did not meet the MCID threshold still reported satisfaction rates comparable to or exceeding those of younger non-achievers. This dissociation between objective functional recovery and subjective satisfaction has been described in the spine literature.^22,23^ Beyond older age, common explanations in the literature for maintaining satisfaction despite missing MCID include private insurance, increased physical independence, psychosocial support factors, and favorable environmental factors.^22,23^ This could also be due to postoperative expectation recalibration, satisfaction derived from disease stabilization rather than functional gain, or a lower preoperative reference point for acceptable postoperative function. The broader literature calls into question whether MCID achievement is a prerequisite for patient satisfaction, a question that extends to an age-stratified context, suggesting the relationship between outcome and satisfaction is fundamentally different across the lifespan. Prior work has similarly demonstrated that MCID thresholds may not be uniform across age groups, and that older patients often derive meaningful satisfaction from outcomes that fall below established benchmarks.^24^

These findings emphasize the importance of preoperative counseling, expectation management, and shared decision-making. Patients in their late forties occupy a critical inflection zone, where they are at or near the P50 threshold for both PROMIS domains, yet present with expectations aligned with younger, higher-achieving counterparts. Surgeons should consider incorporating age-adjusted outcome probabilities into preoperative discussions, particularly for this demographic with the widest expectation-achievement gap. In counseling older patients, surgeons may want to orient surgical goals around symptom stabilization and risk reduction, rather than large objective improvements, in an effort to better align expectations with achievable outcomes. The PROMIS CAT and expected outcome assessment framework employed in this study provides a scalable, efficient infrastructure for embedding this type of expectation management into routine preoperative workflows.

This study had several limitations that merit consideration. As with all retrospective reviews, the study was prone to recall bias, and selection bias may have been introduced if patients lost to follow-up differed systematically from those who completed all necessary surveys. The small final cohort of 129 patients limited statistical power for subgroup analysis, particularly in terms of satisfaction analysis where non-achiever subgroups were small. Additionally, this study was conducted at a single tertiary academic medical center in a metropolitan area, and data socioeconomic status, education level, and insurance type were not collected. These sociodemographic and cultural factors may independently influence pain perception, PROMIS reporting, and preoperative expectations, and their absence limits our ability to fully characterize the expectation-achievement gap and to generalize these findings to more socioeconomically, culturally, and geographically diverse populations. Furthermore, our multivariable models did not include several factors previously associated with reduced likelihood of achieving MCID, including baseline symptom severity and duration, opioid use, depression, and comorbidity burden, several of which may be more prevalent among older patients. Residual confounding from these unmeasured factors cannot be excluded, and the association between age and MCID achievement observed here should be interpreted as independent of the covariates included in our models rather than as evidence that age acts in isolation from these broader clinical and psychosocial contributors. Additionally, PROMIS T-scores are normed to the general U.S. adult population and do not account for the fact that population-based “normal” function and pain interference scores decline with age. Consequently, some portion of the observed age-related decline in MCID achievement may reflect this normative shift rather than a true age-related deficit in surgical benefit. The MCID threshold of 8 absolute value points allowed for uniform comparison across all groups, however there is little literature to explain MCID fluctuations by age in spine patients, especially for PROMIS. While the younger cohort of patients readily achieved MCID, perhaps the older patients should have been compared to a relatively adjusted MCID benchmark. The preoperative counseling conversations between treating surgeons and patients were not standardized, which may have introduced variability in patient expectations that could not be fully accounted for in the analysis. Lastly, follow-up of one year provides a meaningful window for assessing early postoperative recovery, but limits durability assessment of outcomes that may continue to improve or decline beyond one year following surgery, particularly in myelopathy patients where neurological recovery can be prolonged.

## Conclusion

Age is a continuous predictor of failure to achieve MCID after elective cervical spine surgery, independent of the covariates assessed in this study, with older age associated with progressively smaller improvements in physical function and pain interference. Despite this, patient expectations did not vary substantially with age, creating a widening gap between anticipated and actual outcomes that was most pronounced in middle age. When a patient enters their late forties, the probability of achieving clinically meaningful improvement declines to approximately 50% for both domains. Older patients who did not achieve MCID nonetheless reported high satisfaction, suggesting that postoperative satisfaction is not solely determined by functional recovery. These findings emphasize the importance of age-specific preoperative counseling and call for reconsideration of whether a single MCID threshold is appropriate across all age groups.

## Data Availability

The data supporting the findings of this study are not publicly available due to institutional privacy regulations but are available from the corresponding author upon reasonable request.*
